# A nanocarrier delivery system of oxaliplatin for glioblastoma: synthesis and cytotoxicity of Fe_3_O_4_@SiO_2_/OXA nanocomposites

**DOI:** 10.1039/d5ra05686a

**Published:** 2025-12-15

**Authors:** José Miguel Montero Vasquez, Emily Gonzalez, Juan Felipe Zapata-Acevedo, Daniel Llamosa Pérez, Indry Milena Saavedra Gaona, Carlos Arturo Parra Vargas, Jahaziel Amaya, Karina Vargas-Sánchez, Mónica Losada-Barragán, Rolando Javier Rincón Ortiz

**Affiliations:** a GIFAM Group, Faculty of Sciences, Universidad Antonio Nariño Cra. 1 Este #47a15 Bogotá D.C. 110231 Colombia; b Grupo de Investigación en Biología Celular y Funcional e Ingeniería de Biomoléculas Cra. 1 Este #47a15 Bogotá D.C. 110231 Colombia; c Laboratorio de Neurofisiología Celular, Grupo de Neurociencia Traslacional, Facultad de Medicina, Universidad de los Andes Bogotá D.C. Colombia; d Grupo Física de Materiales, Escuela de Física, Universidad Pedagógica y Tecnológica de Colombia Av. Central del Norte #39-115 Tunja Boyacá 150003 Colombia; e GIBICS Group, Faculty of Sciences, Universidad Antonio Nariño Cra. 1 Este #47a15 Bogotá D.C. 110231 Colombia rolricon@uan.edu.co

## Abstract

Among the available treatments for glioblastoma multiforme (GBM), chemotherapy is the most widely used because anticancer drugs can help shrink the tumour. Unfortunately, these drugs present several problems, such as the resistance of cancer cells, low specificity and secondary effects. Using nanocarriers for these drugs is a novel strategy that can reduce the side effects associated with chemotherapy, thereby enabling treatments that are less taxing on the body. In this study, nanocarriers based on superparamagnetic iron nanoparticles coated with silicon oxide were prepared, and the surface of the nanocomposite was functionalised with oxaliplatin, a drug commonly used in chemotherapy. FTIR, XRD, UV-Vis, TEM, and VSM techniques were employed for compositional, structural, morphological, and magnetic characterisation. FTIR confirmed the presence of iron oxide, silica, and oxaliplatin in the nanocomposites, while XRD and TEM analyses revealed the crystalline structure and size distribution of the nanoparticles. UV-Vis spectroscopy indicated the adsorption of oxaliplatin onto the nanoparticle surface, with a gradual increase in adsorption over time. The cytotoxicity of Fe_3_O_4_@SiO_2_/OXA was also evaluated against T98G glioblastoma and BHK-21 non-tumoral cell lines using MTT assays. Fe_3_O_4_ and Fe_3_O_4_@SiO_2_ alone showed minimal cytotoxicity, while the functionalised nanocomposite demonstrated a significant reduction in cell viability, particularly at higher concentrations (IC_50_ of 15 ppm at 72 h), compared to free oxaliplatin. This enhanced cytotoxicity was attributed to the adsorption of oxaliplatin on the nanoparticle surface, amplifying its activity and indicating that functionalisation with oxaliplatin is crucial for the observed therapeutic effects. Fluorescence microscopy and TEM analyses revealed that the Fe_3_O_4_@SiO_2_/OXA nanoparticles were effectively internalised by T98G cells, accumulating primarily in the perinuclear region. Ultrastructural changes, such as mitochondrial swelling, were observed in the treated cells, suggesting that the nanoparticles may induce cellular damage upon internalisation. These results suggest that the nanoparticles enter cells *via* endocytic pathways, potentially enhancing the therapeutic effects of oxaliplatin through more efficient intracellular drug delivery. These findings highlight the potential of Fe_3_O_4_@SiO_2_/OXA as a promising nanocarrier for targeted cancer therapy, with further research needed to elucidate the precise mechanisms of drug release and action within the cell.

## Abstract 2

Among the available treatments for glioblastoma multiforme (GBM), chemotherapy is the most widely used because anticancer drugs can help shrink the tumour.

## Graphical abstract

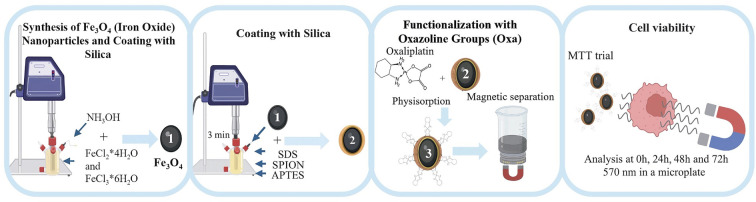

## 1. Introduction

Cancer is one of the leading causes of death in the world. In 2020, almost 10 million deaths were attributed to this disease. Breast, lung, colon and rectum, prostate, skin, and gastric cancers^1^ are among the most common types of cancer at a global level. Additionally, rare cancers with high mortality rates, such as glioblastoma (GBM), remain a major challenge. GBM has an incidence between 3 and 5 cases per 100 000 inhabitants per year worldwide and a survival rate of less than one year after cancer diagnosis. This poor diagnosis is linked to limitations in treatment, largely due to the tumour's location, age-related constraints in patients,^2^ and the scarcity of adequate studies evidencing significant benefits from available therapies.^3^ Additionally, GBM is composed of astrocytes, which have a high chemoresistance due to the expression of proteins from the ABC transporter family. These transporters facilitate the elimination of various metabolites and xenobiotics, among which are chemotherapeutic drugs. This makes the tumour cells more resistant to treatment (making the tumour challenging to treat).^4^

GBM treatments include surgery, as well as immuno-, radio-, and chemotherapies. However, depending on the type of treatment and the location of GBM, these have several disadvantages. Nevertheless, chemotherapy is still one of the most significant references in cancer treatment due to its diverse alternatives against different types of cancers and therapies.^5^

There are currently three strategies for using anticancer drugs: (i) combining different drugs to enhance their effect and improve treatment; (ii) increasing the drug dose; (iii) administering anticancer drugs alongside other techniques such as surgery or radiation.^6^ However, these methodologies do not lessen the burden on the body and can lead to adverse effects because they also damage healthy tissues. Patients who survive treatment also tend to metastasise, a significant problem. Tumour recurrence can lead to chemotherapy limitations such as resistance, increased toxicity, and multiple side effects. The FDA has approved oxaliplatin for the clinical treatment of cancer. It acts as a cytotoxic agent in platinum-based chemotherapy regimens, often in combination with other chemotherapy drugs.^7^

The biochemical industry has been evaluating the use of nanomaterials as nanocarriers due to their advantages over non-functionalized pharmaceutical products used in traditional therapeutics. Due to their small size, nanoparticles have different physical properties, offering an excellent surface area/volume ratio.^8^ Nanostructured materials additionally allow the use of lower dosages, reduce toxicity and are biodegradable.^9^ Their synthesis is economical and easy to incorporate in the pharmaceutical industry, specifically for administering targeted drugs. The superparamagnetic nature of iron nanoparticles is evidenced by their lack of any hysteresis,^10^ that is, they do not maintain spin alignment once the external magnetic field is removed.^11^ These materials are interesting because they exhibit low toxicity and can be easily directed/focused using external magnetic fields. For this reason, the pharmaceutical industry has increased the use of nanotechnological products.^12^

The synthesis process plays a fundamental role in ensuring that the nanocomposite to be produced encompasses these properties. Among the various types of syntheses, biological synthesis uses natural materials and therefore requires minimal amounts of environmentally harmful reagents; however, the synthesis times are often long.^13,14^ Physical syntheses are also characterised by low performance and environmentally friendly processes, but their main problems are releasing large amounts of energy and their synthesis times.^15,16^ Furthermore, we have chemical synthesis that uses simple methodologies but many toxic chemicals.^17^

There has been increased interest in ultrasonic cavitation among chemical methodologies in recent years. This synthesis methodology uses acoustic waves to produce materials at the nanometer scale. In general terms, the cavitation generated is the growth and collapse of bubbles within the liquid. The advantages of this synthesis methodology compared with those described above are its energy efficiency, increased reaction speed, and increased performance without neglecting its economic viability.^18^ Another aspect to be highlighted is the optimisation of the properties of the nanoparticles by modifying the synthesis parameters.^19^ The use of ultrasonically assisted synthesis routes improves the magnetic properties of the nanoparticles produced because it improves the sizes and distribution of the nanoparticles by avoiding the formation of aggregates, which directly affects the development of magnetic or superparamagnetic properties based on the size of the domain.^20^

The increase in the drug payload has enabled the implementation of nanoparticles as a transport agent for cancer drugs.^21^ Consequently, this has led to an increase in the survival rate. Nanocarriers vary in size and morphology, with larger ones favouring drug adsorption and smaller ones promoting better drug penetration into the tumour.^10^ Additionally, spherical morphologies^22^ improve the internalisation of nanocarriers.^10^ In addition to their specific properties, nanoparticles with magnetic properties favour properties such as drug penetration and retention to prevent them from stagnating in the various tissues present.^23^

Among the nanoparticles mentioned above as nanocarriers, superparamagnetic iron oxide nanoparticles (SPIONs) provide properties of great interest for tumour treatment, such as the use of magnetic hyperthermia generated by SPIONs; this gives us the ability to alter the metabolism of thermal shock proteins in the tumour, resulting in cell degradation and cell apoptosis, using an external magnetic field. Additionally, SPIONs can also be used as drug nanocarriers. This is particularly advantageous because it allows for reducing the pharmaceutical load (that is currently used), prevents cell proliferation by enabling drug interaction with cellular DNA, decreases the adverse effects of chemotherapy on non-cancerous cells, improves biocompatibility, and reduces nanoparticle aggregation in biological systems.^24^

The present research proposes to develop a nanocomposite with a superparamagnetic core of iron oxide coated with silica as a potential nanocarrier of drugs for treating GBM through targeted therapy. Achieving this goal begins with synthesising Fe_3_O_4_ nanoparticles coated with SiO_2_ by coprecipitating iron salts, assisted by ultrasonic cavitation. Subsequently, the synthesised nanocomposite was functionalised with the chemotherapeutic drug, oxaliplatin (Fe_3_O_4_@SiO_2_/OXA). Oxaliplatin is a broad-spectrum drug because its primary mechanism of action is to inhibit the synthesis of DNA and RNA, generating covalent metallic adducts with cellular DNA. Oxaliplatin is mainly used because these adducts make error-repairing protein binding to DNA more complex than other platinum pharmaceuticals, such as cisplatin and carboplatin.^25^

Microscopic, spectroscopic, and magnetic techniques were used to characterise the nanocomposite's structural, morphological, and magnetic properties. Finally, the cytotoxic properties exhibited by the Fe_3_O_4_@SiO_2_/OXA nanocomposite on glioblastoma (T98G) and fibroblast (BHK-21) cell lines were evaluated using MTT tests.

## 2. Materials and methods

### 2.1. Materials

The reagents used for the synthesis of nanoparticles were iron chloride(ii) tetrahydrate (FeCl_2_·4H_2_O), iron chloride(iii) hexahydrate (FeCl_3_·6H_2_O), (3-aminopropyl) triethoxysilane (APTES), sodium dodecyl sulfate (SDS) and ammonium hydroxide (NH_3_OH). Type I water was used as the solvent/suspension throughout the synthesis process. The anticancer drug used was oxaliplatin (C_8_H_14_N_2_O_4_Pt, OXA).

Microscopic, spectroscopic, and magnetic techniques were used for structural, morphological, and magnetic characterisation of the nanocomposite. Finally, the cytotoxic properties of the Fe_3_O_4_@SiO_2_/OXA nanocomposite towards glioblastoma (T98G) and fibroblast (BHK-21) cell lines were evaluated using MTT tests. For evaluation by MTT (3-(4,5-dimethylthiazol-2-yl)-2,5-diphenyltetrazolium bromide), the following reagents were used: cell culture medium DMEM (Gibco Dulbecco's Modified Eagle Medium, BioWhittaker), fetal bovine serum (Biowest) for cell culture, phosphate-buffered saline (PBS, BioWhittaker), trypsin (Veisene), MTT (Trevigen), and DMSO (Dimethyl sulfoxide, BioBasic). The T98G cell line (fibroblast-like cells isolated from a glioblastoma multiforme, CRL-1690) was obtained from the American Type Culture Collection (ATCC, Manassas, VA, USA). The T98G cell culture represents cancerous glioblastoma, while the kidney fibroblasts (BHK-21) serve as the healthy cell line.

### 2.2. Nanoparticle synthesis

#### 2.2.1. Synthesis of magnetic nanoparticles (Fe_3_O_4_)

**Fig. 1:**
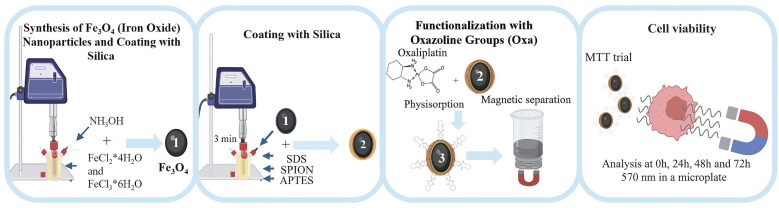
Representative synthesis scheme of the Fe_3_O_4_@SiO_2_/OXA nanocomposite as an anticancer drug delivery system for treating glioblastoma.

The synthesis of Fe_3_O_4_ nanoparticles was carried out by the direct ultrasonically assisted coprecipitation method using Hielscher UP400ST ultrasonicator equipment (400 W, 24 kHz), working in a pulsing cycle of 0.9 s on and 0.1 s off and adjusted to an ultrasonic power of 60% of the maximum power (400 W). The iron salts (FeCl_2_·4H_2_O and FeCl_3_·6H_2_O) were used in solution with a molar ratio of 1 : 2, respectively; the methodology is briefly described below. 516.88 mg of FeCl_2_·4H_2_O and 1408.04 mg of FeCl_3_·6H_2_O were dissolved in 100 mL of type 1 water. Once the solution of the iron salts was properly homogenised, they were coprecipitated with a solution of 100 mL of NH_3_OH at 25%; this process was carried out by dripping until the complete addition of 100 mL of the reducing solution, controlled by a BT101F peristaltic pump. The entire synthesis process was irradiated with ultrasound (US) and carried out inside a water-ice bath whose purpose was to control the temperature of the reaction medium, which reached a maximum temperature of 35 °C. Neodymium magnets were employed to precipitate the final colloidal dispersion magnetically, washing the precipitate obtained thrice using Type I water and discarding the supernatant. The SPIONs are resuspended in 100 mL of Type I water and divided into two fractions. The first fraction of 33.3 mL was used to characterise the uncoated nanoparticles and placed in a drying oven (Thermo Scientific) at 40 °C for 48 h. The second fraction of 66.6 mL was coated with APTES to obtain the nanocomposite Fe_3_O_4_@SiO_2,_ as illustrated in Fig. 1.

#### 2.2.2. Coating Fe_3_O_4_ nanoparticles with SiO_2_ (APTES)

For coating the Fe_3_O_4_ iron oxide nanoparticles, a solution of 720 mg of SDS was first prepared in 100 mL of type I water, which helps the dispersion of the SPIONs obtained in the previous step and thus avoids their aggregation. The mixture is homogenised using an indirect ultrasonic bath. Once homogenised, the 66.6 mL of SPIONs was suddenly added; this process was carried out using direct ultrasound for 3 min; then, by dripping, 3.2 mL of our SiO_2_ precursor (APTES) was added, and the nanocomposite produced was washed thrice. Finally, the nanocomposite was dried at 40 °C for two days.

#### 2.2.3. Functionalization of the nanocomposite Fe_3_O_4_@SiO_2_ with the drug oxaliplatin (OXA) to form the Fe_3_O_4_@SiO_2_/OXA system

The adsorption capacity of the nanoparticles against the drug of interest was determined through the physisorption of oxaliplatin. This physisorption test was performed for the assembly of cell viability assays. 200 µg of nanoparticles were taken and resuspended in 8 µL of the initial solution of oxaliplatin (which contains a concentration of 100 mg in 40 mL); these have 200 µg of oxaliplatin. This solution was homogenised for 20 min in a vortex stirrer, then dried at 25 °C for one day. Once the drying process had finished, the magnetic separation of the nanoparticles was carried out; this obtained only our Fe_3_O_4_@SiO_2_/OXA system and the excess traces of oxaliplatin that were not physisorbed on the surface of the nanocomposite were discarded. The nanocomposite with magnetic properties was resuspended in DEMEM. The final product was irradiated with UV light for 20 min to sterilise the suspension and use it in cell viability tests.

### 2.3. Characterization of the nanocomposite

#### 2.3.1. Fourier transform infrared spectroscopy (FTIR)

Infrared spectroscopy with Fourier transform spectrum measurement was used to establish the bonds in the Fe_3_O_4_ nanoparticles and Fe_3_O_4_@SiO_2_. 30 mg of powder was used for each sample of Fe_3_O_4_, Fe_3_O_4_@SiO_2,_ and Fe_3_O_4_@SiO_2_/OXA. A Bruker brand Alpha Platinum series FTIR spectrophotometer recorded the IR region of 4000–500 cm^−1^.

#### 2.3.2. X-ray diffraction (XRD)

An Analytical Minipalm 2 equipped with an X-ray source with a high-stability copper anode was used. This diffractometer works with Bragg–Brentano geometry. Samples of 30 mg of Fe_3_O_4_, Fe_3_O_4_@SiO_2,_ and Fe_3_O_4_@SiO_2_/OXA powder, previously dried and milled, were analysed. The measurements were made with an angular pitch of 2*θ*° and an angular range of 15° to 80° in 2*θ*.

#### 2.3.3. UV-Vis spectroscopy

The equipment employed was a Merck Spectroquant® Prove 600 from 200 to 500 nm with a step size of 1.0 nm in 10 mm cuvettes. All experiments were performed three times under ambient conditions.

#### 2.3.4. Transmission electron microscopy (TEM)

For this analysis, an FEI Tecnai G2 F20 transmission electron microscope operated at 200 kV was used to obtain images at 145 000×. The samples were prepared by diluting 1 mL of Fe_3_O_4_, Fe_3_O_4_@SiO_2,_ and Fe_3_O_4_@SiO_2_/OXA in type I water using ultrasonic irradiation. Finally, a drop was deposited on a copper grid coated with a lacey-type carbon film.

#### 2.3.5. Vibrating sample magnetometry (VSM)

The magnetic characterisation of the nanoparticles was performed by analysing the magnetic hysteresis curve obtained by vibrating sample magnetometry (VSM). A Quantum Design magnetometer (VersaLab TM) was used at 300 K, with an applied field of −3T to 3T. 30 mg powder was used for each sample of Fe_3_O_4_, Fe_3_O_4_@SiO_2_ and Fe_3_O_4_@SiO_2_/OXA.

### 2.4. Determination of the concentration of oxaliplatin in the nanocomposite Fe_3_O_4_@SiO_2_

For this test, a calibration curve was first performed to obtain the function of the line corresponding to the Lambert–Beer equation, with an initial concentration of 2.5 mg mL^−1^ of oxaliplatin and serial dilutions of the same until a final concentration of 0.039 mg mL^−1^ of oxaliplatin was obtained. Subsequently, suspensions of the nanocomposite (Fe_3_O_4_@SiO_2_) were measured in the presence of the drug with intervals of 0 min, 15 min, and 30 min, with a concentration of 1 mg of nanocomposite and 2.5 mg of oxaliplatin (1 mL). For 0 min, the spectrum of the drug alone, equivalent to zero drug adsorption by the nanocomposite, was measured. For 15 min, the nanocomposite was homogenised in the presence of the drug using a vortex. Then, the nanoparticles were decanted with the help of a magnetic field, and the supernatant was removed, which was measured in a UV-Vis spectrophotometer (Spectroquant® Prove 600). The nanocomposite was resuspended again in 1 mL of oxaliplatin and placed in the vortex for 30 min; after 30 min, the nanoparticles were decanted, and the supernatant of the sample was measured again. This was done to establish the most suitable interaction time for the physisorption of oxaliplatin by the nanocomposite. All the targets used corresponded to type I water.

### 2.5. Cell viability assay in glioblastoma multiforme and fibroblasts

The T98G (fibroblast-like cells isolated from a glioblastoma multiforme, CRL-1690) and BHK-21 (Baby Hamster Kidney-21, ATCC-CCL 10) cell lines were used for cytotoxicity assays. They were maintained in Dulbecco's modified minimal essential medium (DMEM – Lonza®, Catalogue No. 12-604Q), supplemented with 10% fetal bovine serum (FBS) (Biowest®, Catalogue No. S18b-500), and 1% penicillin-streptomycin antibiotic solution (Lonza®, Catalog No. 17-602F) and incubated at a temperature of 37 °C with a 5% CO_2_ atmosphere.

MTT tests were carried out to evaluate the cytotoxicity of the SPIONs, Fe_3_O_4_@SiO_2_, Fe_3_O_4_@SiO_2_/OXA, and oxaliplatin alone. The T98G cell line was seeded in a 96-well plate at a cell density of 10 000 cells per well and incubated for 24 h. T98G cells were incubated with Fe_3_O_4_, Fe_3_O_4_@SiO_2_, Fe_3_O_4_@SiO_2_/OXA, and OXA at 5, 25, 50, 100, and 200 ppm for 0, 24, 48, and 72 h. DMSO 5% and standard culture medium were included as controls. Three replicates were set up in each group. After the incubation time, 10 µL of MTT reagent was added to each well, and the plate was incubated for four hours. The medium was discarded, and 100 µL of DMSO was added, allowing the incubation of the plate for an additional 20 min. The absorbance was read at a wavelength of 570 nm in a microplate reader (Multiskan™ FC, Thermo Scientific). The percentage viability was calculated compared to untreated cells. The IC_50_ was calculated through a normal log distribution and then a nonlinear regression between the logarithm of the inhibitor and the response given by the stimuli (in % of viability), which was 15 ppm at the time of 72 h. This concentration was used to evaluate the cytotoxic properties of Fe_3_O_4_ and Fe_3_O_4_@SiO_2_. All assays were performed in triplicate. To analyse the effects of the nanocomposites on the cell viability of the BHK-21 line, the IC_50_ calculated in the tests with T98G cells (with oxaliplatin) was used.

### 2.6. Immunofluorescence

T98G cells were seeded in 24-well plates on coverslips coated with poly-l-lysine (2 µg cm^−2^) at a density of 50 000 cells per well in a complete medium. The cells were allowed to grow until 80% confluence was reached. Subsequently, Fe_3_O_4_, Fe_3_O_4_-APTES, and Fe_3_O_4_-APTES-oxaliplatin nanoparticles were added at a final concentration of 15 ppm in fresh medium. The cells were incubated for 72 h at 37 °C in a 5% CO_2_ atmosphere with gentle agitation. After incubation, the medium was removed, and the cells were washed with sterile PBS. Fixation was performed using 500 µL of 4% paraformaldehyde per well for 30 min at room temperature, followed by washing with PBS.

The cells were saturated with 3% goat serum and permeabilised with 0.3% Triton X-100 in PBS for 30 min. The primary antibody, anti-GFAP rabbit (ref: GTX108711, GeneTex), was then added and incubated for 24 h at 4 °C. Following this, the cells were washed with PBS, and the secondary antibody, goat anti-rabbit IgG Alexa Fluor 488 (ref: ab150077, Abcam), was added for 1 h in the dark with gentle shaking. The nuclei were labelled with DAPI (ref: F6057, Sigma-Aldrich).

Immunofluorescence images were captured using an HC PL FLUOTAR L 40×/0.60 DRY objective on a Leica DMi8 microscope (Leica Microsystems, Germany) with Leica Application Suite X (LAS X) software (version 3.7.5.24914). The following camera settings were utilised: Camera (DFC7000T-0056173616), Bin1x1 Format (1920x1440), Scanning (8-bit), Quality Mode (40 MHz), Color Capture Mode (Composite), Live Bin1x1 Format (1920x1440), TXR (EX: 540–580, DC: 585, EM: 592–668), DAPI (EX: 327–383, DC: 400, EM: 435–485), and FITC (EX: 460–500, DC: 505, EM: 512–542). Three random images were taken per field. ImageJ version 1.53q (Java 1.8.0_172 (64-bit), NIH, USA) was used for image analysis. The Pearson correlation coefficient (PCC) was calculated using the Just Another Colocalization Plugin (JACoP) in ImageJ for colocalisation and correlation analyses.

### 2.7. Transmission electron microscopy (TEM)

T98G cells were cultured in 6-well plates at a density of 100 000 cells per well until reaching 80% confluence. They were then treated with APTES-conjugated iron nanoparticles and oxaliplatin at a concentration of 15 ppm and incubated for 3 d at 37 °C and 5% CO_2_, protected from light, with gentle shaking. After incubation, the cells were trypsinized and centrifuged at 1000 rpm for 10 min. The cell pellet was resuspended in 2.5% glutaraldehyde and centrifuged at 13 000 rpm for 3 min. Post-fixation was performed with 1% osmium tetroxide in water (2 h, 4 °C), followed by pre-impregnation with 3% uranyl acetate (1 h, room temperature). Dehydration was performed using ethanol gradients (50%, 70%, 90%, 100%, 100%; 10 min each), followed by acetone–ethanol (1 : 1, 15 min) and pure acetone (15 min). Embedding in SPURR epoxy resin was performed in stages: Spurr Resin-acetone mixtures (2 : 1 and 1 : 1, 1 h each), pure Spurr Resin (2 h), and polymerisation (12 h, 72 °C). 130 nm-thick sections were obtained using a Leica EM UC7 ultramicrotome and counterstained with 6% uranyl acetate and lead citrate. The samples were examined on a JEOL 1400 plus TEM, capturing images with a Gatan Orius CCD camera.

### 2.8. Statistical analysis

Statistical analysis was performed in ImageJ, OriginPro® 2021b, and GraphPad Prism version 8.0.1. Differences between samples and the control were assessed using the Student's *t*-test. A *p*-value <0.05 was considered statistically significant.

## 3. Results and discussion

### 3.1. Characterization techniques

Below are the results of the morphological, structural, crystalline, chemical, and magnetic properties of Fe_3_O_4_, Fe_3_O_4_@SiO_2,_ and Fe_3_O_4_@SiO_2_/OXA, in addition to presenting the results of cell viability in the T98G and BHK-21 cell lines.

#### 3.1.1. Fourier transform infrared spectroscopy (FTIR)

**Fig. 2:**
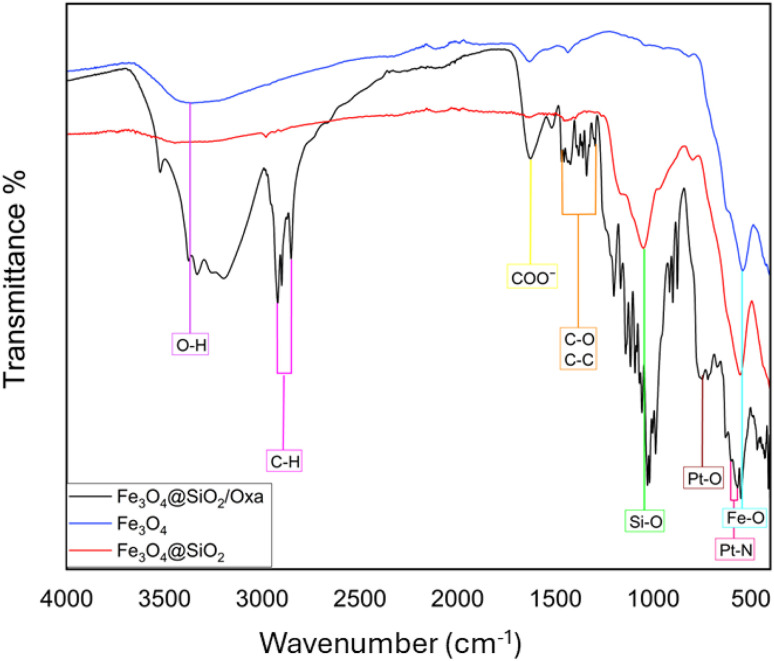
FTIR spectrometry of Fe_3_O_4_, Fe_3_O_4_@SiO_2,_ and Fe_3_O_4_@SiO_2_ functionalised with oxaliplatin.

An FTIR analysis was performed for the Fe_3_O_4_, Fe_3_O_4_@SiO_2_, and Fe_3_O_4_@SiO_2_/OXA samples, as shown in Fig. 2. This shows the vibrations of the bonds in the nanoparticles and oxaliplatin. To confirm the presence of the magnetic core of the nanocomposite, a characteristic band for the vibration of the Fe–O bond close to 500 cm^−1^ is observed, thus indicating the presence of iron oxide. For the coating of the nanoparticles with SiO_2_, the characteristic absorption bands were observed at 1000 cm^−1^, associated with the vibration of asymmetric and symmetric stretching of the Si–O bond. Additionally, bands observed at 521 cm^−1^ and 575 cm^−1^ correspond to vibrations of the Pt–N and Pt–O bonds, confirming the presence of the drug oxaliplatin in the nanocomposite. The bands centred at 1300 cm^−1^ correspond to the oxalate ligand group, establishing the presence of the drug. The absorption band centred at 1666 cm^−1^ confirms the presence of oxaliplatin in the nanocomposite of Fe_3_O_4_@SiO_2_. The peak at 3400 cm^−1^ is due to the stretching vibrations of the O–H bond, associated with the presence of water adsorbed on the silica surface. Finally, the characteristic signals of the C–H bonds were observed at 2900 cm^−1^ and 2860 cm^−1^.^26–29^

#### 3.1.2. X-ray diffraction (XRD)

**Fig. 3:**
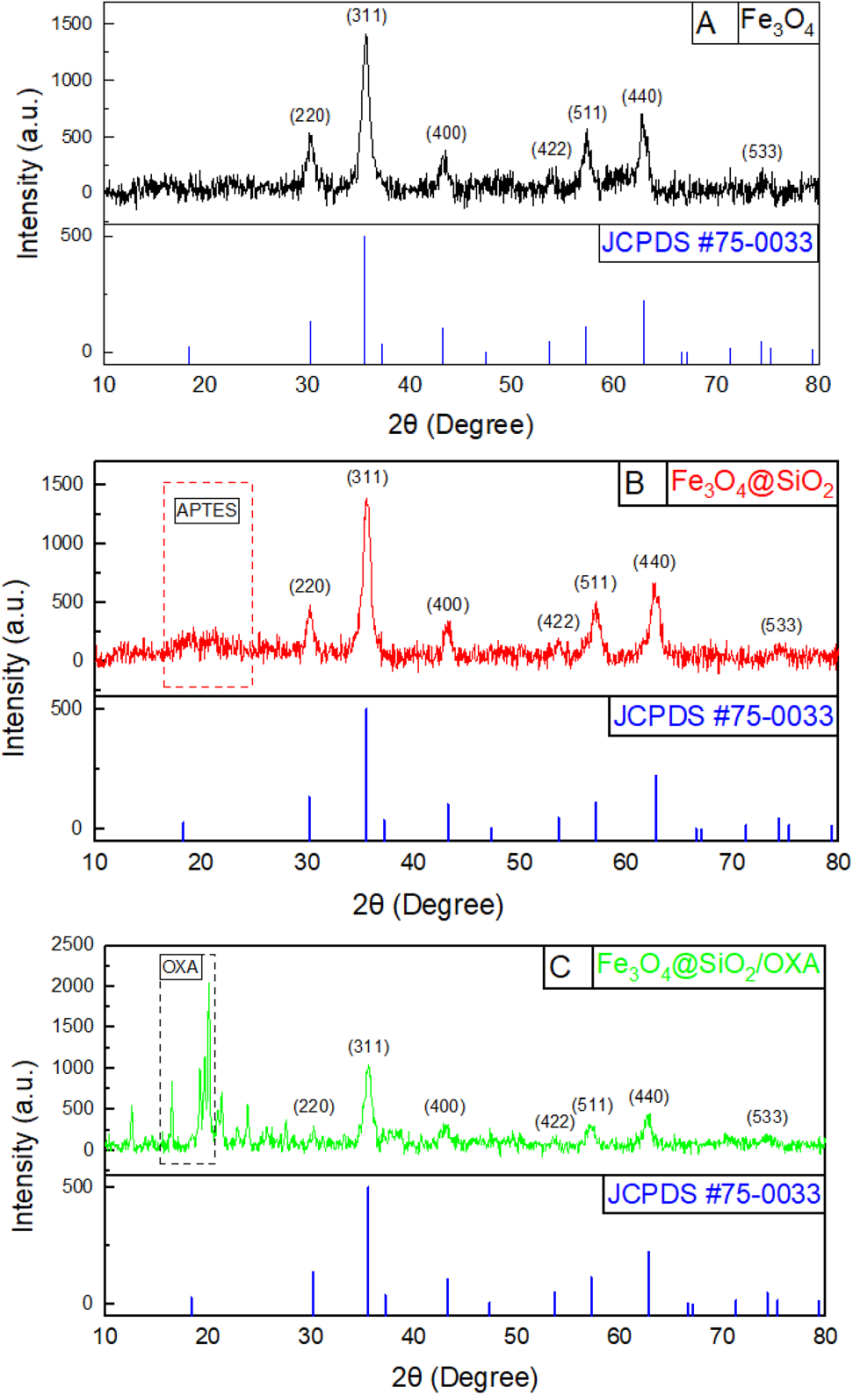
X-ray diffraction (XRD) corresponding to Fe_3_O_4_ (A), Fe_3_O_4_@SiO_2_ (B), and Fe_3_O_4_@SiO_2_ nanoparticles functionalised with oxaliplatin (C).

The diffractogram of the Fe_3_O_4_, Fe_3_O_4_@SiO_2_, and Fe_3_O_4_@SiO_2_/OXA nanoparticles is shown in Fig. 3. The most intense peaks correspond to the characteristic (220), (311), (400), (422), (511), (440) and (533) planes of the cubic crystal structure of magnetite reverse spinel, corresponding to the crystallographic card (JCPDS card #75-0033). To determine this, the interplanar distances are compared and evaluated to determine whether they correspond to their assignment on a certified crystallographic card. In the case of Fe_3_O_4_@SiO_2_, an increase in the signal between 15° and 30° can be observed (red box), confirming the presence of amorphous silica.^30^ Additionally, when comparing the peaks with the crystallographic card, it is observed that they do not present any shift, which indicates that the crystal structure of the magnetite nucleus is maintained after coating Fe_3_O_4_ with SiO_2_, as well as after functionalisation with oxaliplatin. However, a decrease in some of the prominent peaks of SiO_2_ compared to iron can be seen due to the amorphous phase presented by silicon oxide. In the case of functionalisation with oxaliplatin, the decrease in the size of the main peaks is even more due to the sugars and other compounds present in the drug, which precipitate amorphously.

With the peaks obtained in XRD, the values of the crystallite size were calculated, as determined using the Scherrer equation, *D* = *Kλ*/*β* cos, where *λ* corresponds to the wavelength of the incident beam, *β* is the average height width of the peak, *θ* is the angle of reflection of the most intense peaks and *K* = 0.89, which is a coefficient dependent on the shape of the crystal, *i.e.* whether the particles are spheroidal, cubic, tetrahedral or octahedral, in addition to the method by which the size of the crystallite is calculated. With this formula and the calculated values of the main peaks of Fe_3_O_4_, Fe_3_O_4_@SiO_2,_ and Fe_3_O_4_@SiO_2_/OXA, the crystallite size was obtained, which was 8.7 nm for Fe_3_O_4_ and 7.4 nm for Fe_3_O_4_@SiO_2_, and due to the shielding of the signals and due to the increasing amorphous phase, the size for Fe_3_O_4_@SiO_2_/OXA could not be calculated.

The diffractogram of Fe_3_O_4_@SiO_2_/OXA showed intense peaks of oxaliplatin at the 2*θ* values of 16.32°, 19.23°, and 20.66° (black box); this is due to its crystalline nature.^31^

#### 3.1.3. Ultraviolet-visible spectroscopy (UV-Vis)

Ultraviolet-visible spectroscopy (UV-Vis) was utilised to investigate the adsorption efficiency of oxaliplatin on the magnetic core (Fe_3_O_4_) and the corresponding nanocomposite (Fe_3_O_4_@SiO_2_). The analysis was conducted within the spectral range of 200–500 nm, specifically targeting the characteristic absorption peak of oxaliplatin at about 300 nm. Both the magnetic core and the nanocomposite were exposed to oxaliplatin for predetermined intervals of 0, 15, and 30 min to assess their adsorption capacities over time.

**Fig. 4:**
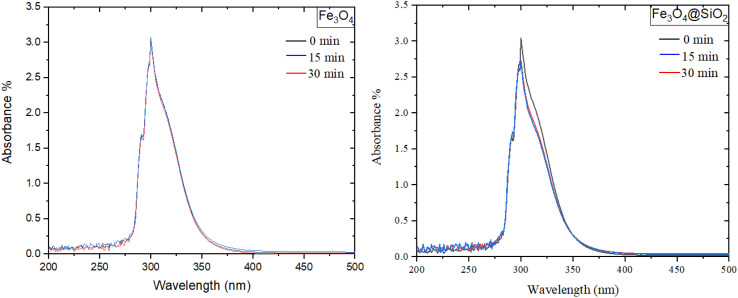
Oxaliplatin absorbance curves in the UV-Vis of Fe_3_O_4_ and Fe_3_O_4_@SiO_2_.

The findings, detailed in Fig. 4, indicate that after 15 min, Fe_3_O_4_ adsorbs approximately 1.8% of oxaliplatin relative to its initial concentration, whereas Fe_3_O_4_@SiO_2_ demonstrates a significantly higher adsorption of 11%. This notable difference underscores the role of the SiO_2_ coating in enhancing the interaction between the nanocomposite and the oxaliplatin molecules. Upon extending the exposure time to 30 min, the magnetic core exhibits negligible adsorption relative to its initial concentration, indicating saturation or minimal interaction capability. In contrast, the Fe_3_O_4_@SiO_2_ nanocomposite achieves cumulative adsorption of 22%, effectively doubling its performance over the previous interval.

These results underscore the superior adsorption properties conferred by the SiO_2_ coating on the magnetic core, likely due to increased surface area and improved chemical compatibility with oxaliplatin. The progressive and sustained adsorption observed in Fe_3_O_4_@SiO_2_ highlights its potential as an efficient carrier for oxaliplatin, with promising applications in targeted drug delivery and other biomedical fields.

#### 3.1.4. Transmission electron microscopy (TEM)

**Fig. 5:**
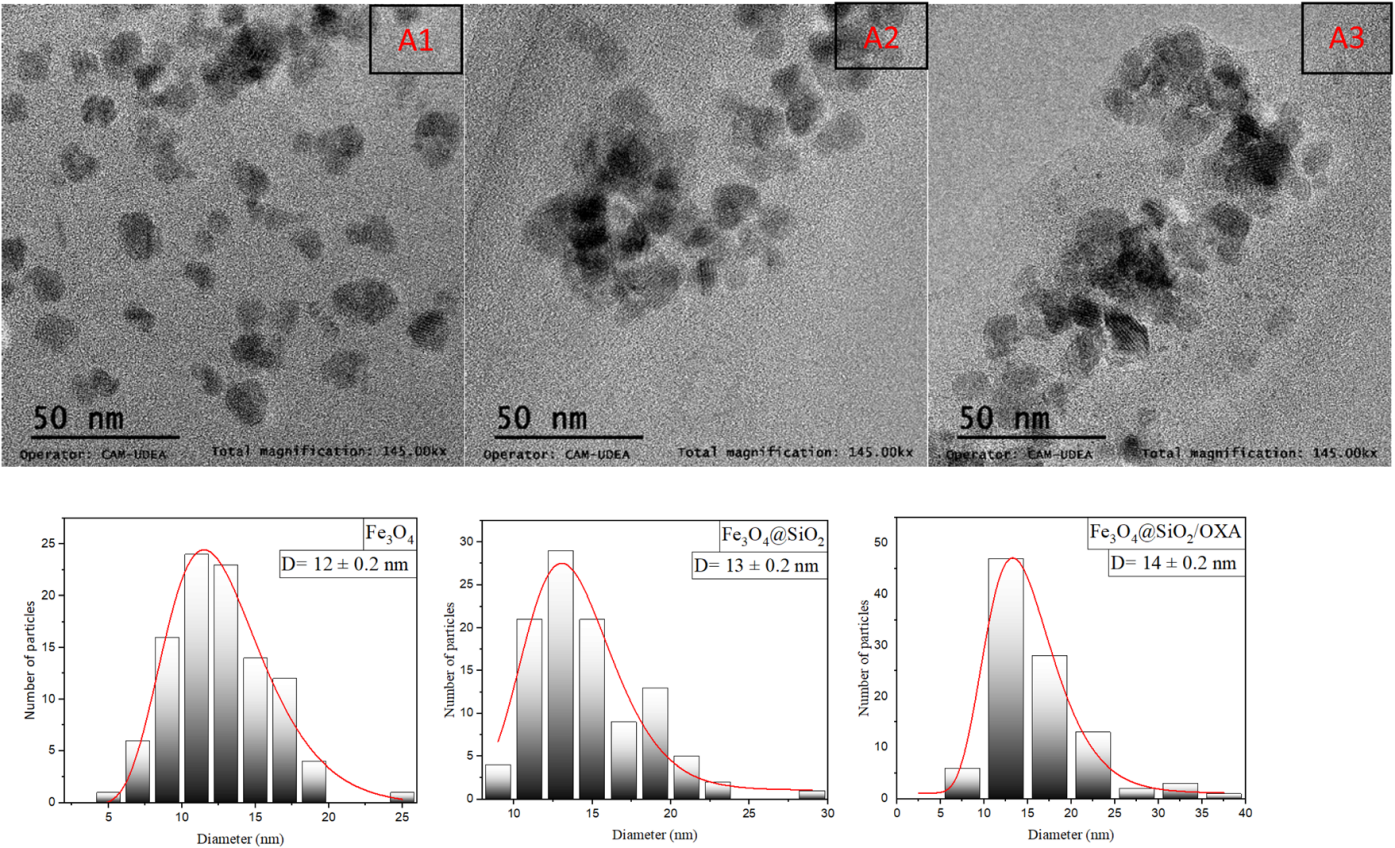
Scanning electron microscopy (TEM) corresponding to Fe_3_O_4_ (A1), Fe_3_O_4_@SiO_2_ (A2), and Fe_3_O_4_@SiO_2_ nanoparticles functionalised with oxaliplatin (A3) and the corresponding size distributions.

TEM was used for morphological characterisation of the nanoparticles to determine the sizes of the nanoparticles and to study the effect of APTES on the size distribution of the nanoparticles, as shown in Fig. 5. To determine the size of the nanocomposites Fe_3_O_4_, Fe_3_O_4_@SiO_2,_ and Fe_3_O_4_@SiO_2_/OXA, the analysis of these images using ImageJ software was performed, with which it was possible to measure the diameters of the nanoparticles obtained. Through a normal log adjustment and a count of 100 nanoparticles from various images given by the team, it was possible to show a gradual increase between the single, coated, and functionalised nanoparticles; this was due to the presence of SiO_2_ on the surface of the iron nanoparticles and the presence of the drug. Based on these results, we can confirm that the iron nanoparticles have a spheroidal morphology and an approximate size of 12 nm; the nanoparticles, once coated with SiO_2_, maintain a spheroidal morphology, but due to the coating, there is evidence of an increase in size to 13 nm. The functionalised nanoparticles continue to present a spheroidal morphology, and the size of the nanoparticles increased to 14 nm. These sizes are of great interest as sizes below 50 nm favour the internalisation of nanoparticles into the target cells. Additionally, their spheroidal morphology is conducive to such internalisation in target cells and through various biological barriers.^32^

##### 3.1.4.1. High-resolution transmission electron microscopy (HRTEM)

**Fig. 6:**
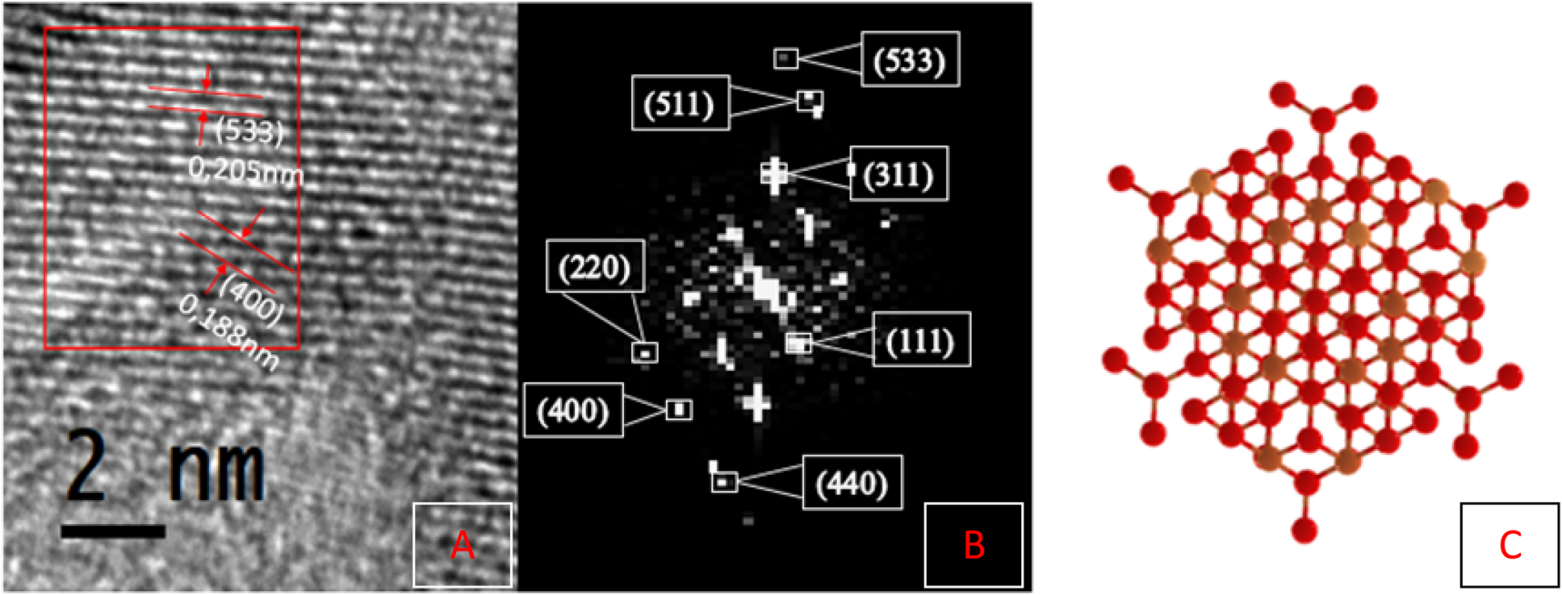
High-resolution transmission electron microscopy (HRTEM) of Fe_3_O_4_: interplanar distances (A), FFT (B), and the crystallographic structure of reverse spinel magnetite (C).

High-resolution electron micrographs (HRTEM) were performed to observe the spatial arrangement of the atoms and thus study the crystal structure of the Fe_3_O_4_ nanoparticles, as shown in Fig. 6. For this, the DigitalMicrograph® software was used, with which it is possible to perform a fast Fourier transform, to calculate the distances within the crystalline planes. Based on these results, the nanoparticles present crystallinity fringes with interplanar distances of 0.205 nm and 0.188 nm for the (533) and (400) planes, as shown in Fig. 6. This indicates that the nanoparticles exhibit high ordering at short and medium ranges. Additionally, the HR-TEM images can be converted into diffraction patterns through the fast Fourier transform (FFT) method for crystallographic analysis (Fig. 6B). From the FFT, the interplanar distances are calculated; these correspond to the (111), (220), (311), (400), (511), (440), and (533) planes, which correspond to the reverse spinel magnetite structure (Fig. 6B), which coincides with the JCPDS #75-003 crystallographic card. To finish using the Materials Project database,^33^ a simulation of the crystallographic structure of reverse spinel magnetite Fe_3_O_4_ was carried out to contrast it with the crystalline structure shown by the synthesised nanoparticles (Fig. 6C). These similarities are seen in the shape and distribution of the atoms, resulting in hexagonal nanostructures typical of magnetite.

To complement the results of HRTEM, selected area electron diffraction (SAED) analysis was carried out to confirm the reverse spinel lattice plane and the planes corresponding to magnetite.

##### 3.1.4.2. Selected area electron diffraction (SAED)

**Fig. 7:**
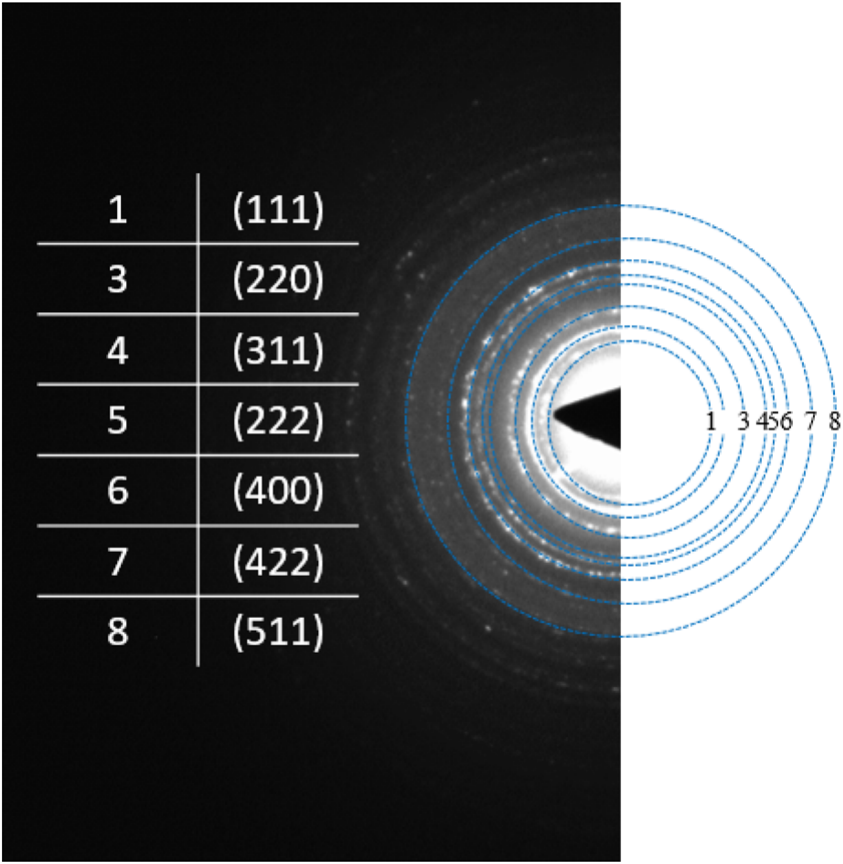
The selected area electron diffraction (SAED) spectrum of Fe_3_O_4_.

The electron patterns of the chosen area exhibit the different rings of the crystalline phase of Fe_3_O_4_, which correlate with the (111), (220), (311), (222), (400), (422) and (511) planes; this indicates that the nanoparticles present a reverse spinel network plane for Fe_3_O_4_ (Fig. 7) (with JCPDS indexed drawings #75-003).^34,35^ This determination was made using the ImageJ imaging software and the equation of the TEM camera 
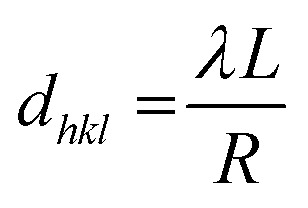
, where *d*_*hkl*_ refers to the interplanar distance, *λL* is the constant of the camera, which is equal to 205.249217 Å, and *R* is the radius of each diffraction ring, to find the interplanar distance.

##### 3.1.4.2. Energy-dispersive X-ray spectroscopy (EDS)

**Fig. 8:**
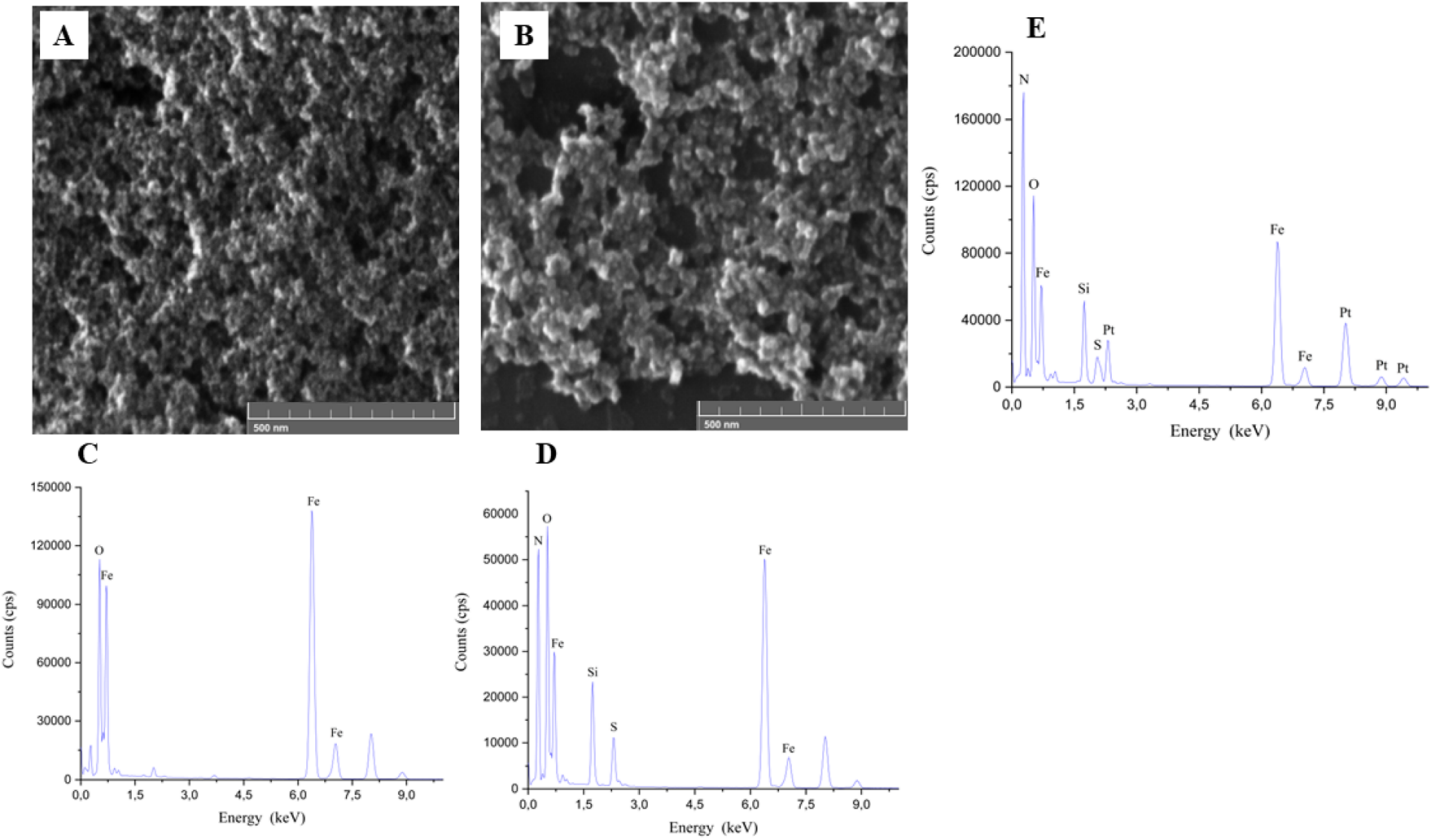
SEM images of (A) Fe_3_O_4_, and (B) Fe_3_O_4_@SiO_2_. X-ray spectrometry (EDS) spectra of (C) Fe_3_O_4_, (D) Fe_3_O_4_@SiO_2_ and (E) Fe_3_O_4_@SiO_2_/OXA nanoparticles.

The analysis of energy dispersive X-ray spectroscopy (EDS) (Fig. 8) gives the semi-quantitative composition of the elements present in the samples of Fe_3_O_4_ (C), Fe_3_O_4_@SiO_2_ (D), and Fe_3_O_4_@SiO_2_/OXA (E). EDS mapping indicates that for the samples of Fe_3_O_4_, the presence of Fe is detected with a percentage composition of 65.34% and the presence of O with 34.66%. These results confirm that only iron and oxygen (corresponding to iron oxide) were present, and there was no contamination during the synthesis of the magnetic core. For the Fe_3_O_4_@SiO_2_ samples, we found the presence of nitrogen, oxygen, iron, silica, and sodium, with a percentage by weight of (1.10%), (35.84%), (46.93%), (5.88%) and (5.88%), respectively; this indicates the presence of our magnetic core (Fe_3_O_4_). In addition to the characteristic peaks of SiO_2_, as there are peaks of Si, and a peak of N belonging to our precursor of silica oxide (APTES (C_9_H_23_NO_3_Si)), we observed the presence of sodium, originating from the nanoparticle dispersant used to prevent aggregation (SDS (NaC_12_H_25_SO_4_)). Finally, the spectrum of the Fe_3_O_4_@SiO_2_/OXA sample shows the appearance of nitrogen (1.89%), oxygen (32.80%), silica (10.22%), sodium (6.69%), iron (38.02%) and platinum (10.38%), which indicates the presence of our nanocomposite Fe_3_O_4_@SiO_2_, in addition to the presence of our drug oxaliplatin, indicated by the peaks present of platinum in the sample and the peaks corresponding to APTES and SDS.

#### 3.1.5. Vibrating sample magnetometry (VSM)

**Fig. 9:**
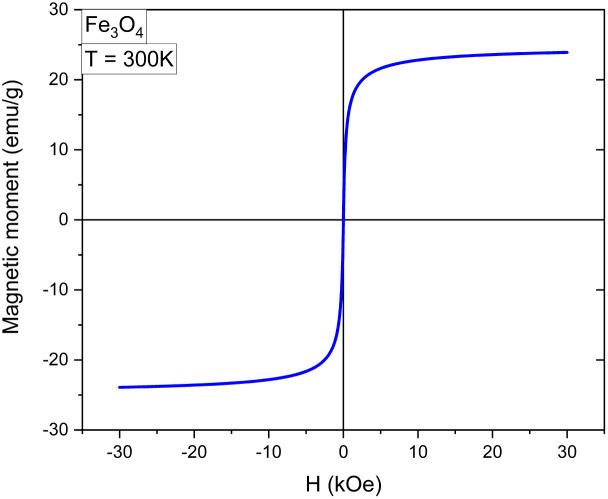
Magnetic characterization curves of the Fe_3_O_4_, Fe_3_O_4_@SiO_2_ and Fe_3_O_4_@SiO_2_/OXA nanoparticles.

An analysis by magnetometry (VSM) was performed to evaluate the magnetic properties of Fe_3_O_4_, Fe_3_O_4_@SiO_2,_ and Fe_3_O_4_@SiO_2_/OXA (Fig. 9). This was done as a function of the magnetic field and at room temperature (300 K), using a magnetic field of −30 kOe to 30 kOe, and was done to determine the presence or absence of hysteresis loops at room temperature, and the magnetisation saturation (Ms) of Fe_3_O_4_, Fe_3_O_4_@SiO_2,_ and Fe_3_O_4_@SiO_2_/OXA was 23.9, 13.2 and 9.9 emu/g, respectively. This decrease in Ms is due to the coating of SiO_2_ and the presence of oxaliplatin in the samples. It may also be due to the dilution of the magnetic material within the volume of the sample when it is coated with the non-magnetic material, *i.e.*, the moment per unit volume (and hence the moment per unit mass) is reduced. Therefore, the saturation magnetisation decreases. However, this decrease did not affect the coercivity values in any of the three samples, which were zero (at room temperature). This indicates that our three samples have superparamagnetic properties, which is of great interest in this work,^36,37^ because our superparamagnetic nanoparticles can be directed to the target tumour using external magnetic fields.

### 3.2. T98G and BHK-21 cell viability assays

**Fig. 10:**
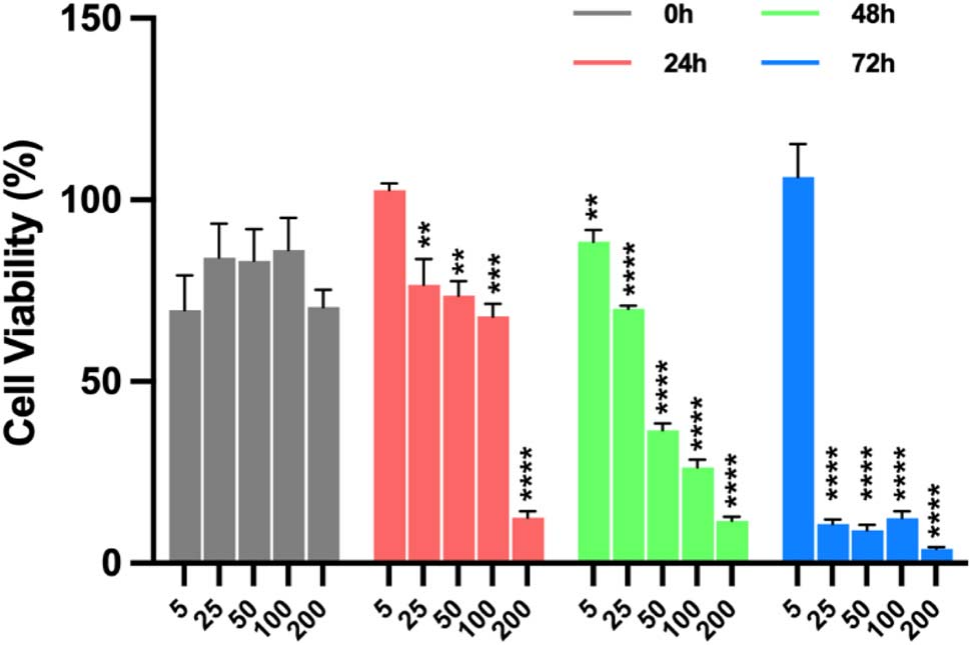
: Percentage of cell viability of the T98G cell line induced by the nanocomposite Fe_3_O_4_@APTES/OXA. Cells were incubated with the nanocomposite Fe_3_O_4_@APTES/OXA at 5, 25, 50, 100 and 200 ppm concentrations for 0, 24, 48, and 72 hours. Data are presented as mean ± standard deviation of the mean SEM (*n* = 3). Data were analysed using a one-way ANOVA with a Dunnett's post-hoc test (*p < 0.1, **p < 0.01, ***p < 0.001, ****p < 0.0001).

**Fig. 11:**
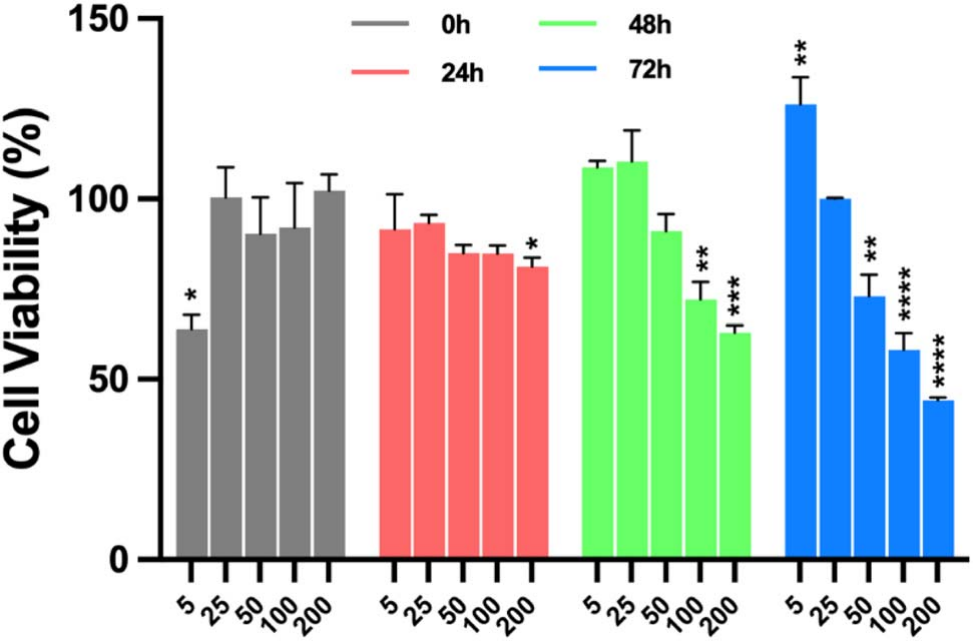
Percentage of cell viability of the T98G cell line induced by oxaliplatin (OXA). Cells were incubated with OXA at a concentration of 5, 25, 50, 100 and 200 ppm for 0, 24, 48, and 72 hours (this was done in triplicate). Data are presented as mean ± standard deviation of the mean SEM (*n* = 3). Data were analysed using a one-way ANOVA with a Dunnett's post-hoc test (*p < 0.1, **p < 0.01, ***p < 0.001, ****p < 0.0001).

**Fig. 12:**
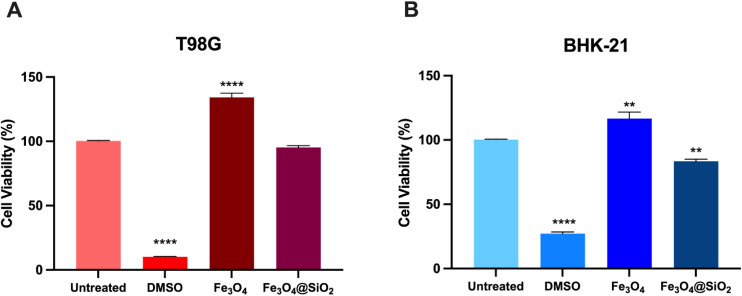
Percentage of cell viability induced by Fe_3_O_4_ and Fe_3_O_4_@SiO_2_ of the T98G cell line and BHK-21 cell line. (A) Glioblastoma cells and (B) fibroblast cells were incubated with Fe_3_O_4_ and Fe_3_O_4_@SiO_2_ at a concentration of 15 ppm for 72 h (IC_50_). Data are presented as mean ± standard deviation of the mean SEM (*n* = 3). Data were analysed using a one-way ANOVA with a Dunnett's post-hoc test (**p < 0.01, ****p < 0.0001).

To assess the impact of nanoparticles functionalised with OXA on cell viability, an MTT assay was performed in which T98G cells were exposed to Fe_3_O_4_@SiO_2_/OXA or OXA at concentrations ranging from 5 to 200 ppm for 0, 24, 48, and 72 hours (Fig. 10, 11 and 12). At 0 h, cell proliferation in the T98G cell line was unaffected by any treatment or concentration of Fe_3_O_4_@SiO_2_/OXA. However, after 24 h, the functionalised nanocomposite exhibited a cell viability of ∼76% at concentrations between 25 and 100 ppm, with an 88% reduction at 200 ppm (*p* < 0.0001). After 48 h, the reduction in viable cells was more pronounced than at 24 h, with viability dropping below 50% at 50, 100, and 200 ppm concentrations (*p* < 0.0001). Notably, at 72 h, all concentrations above five ppm led to a significant reduction in cell viability of approximately 90% (Fig. 10). Thus, Fe_3_O_4_@SiO_2_/OXA exhibited the highest cytotoxicity, with the lowest IC_50_ value (15 ppm) at 72 h compared to the other time points.

In contrast, oxaliplatin exhibited a less pronounced cytotoxic effect in T98G cells than Fe_3_O_4_@SiO_2_/OXA (Fig. 11). At 24 h, just the highest concentration tested (200 ppm) induced a significant reduction of 19% in cell viability compared to the untreated group (*p* = 0.0402). At 48 hours of treatment, oxaliplatin at 100 ppm (*p* = 0.0022) and 200 ppm (*p* = 0.0002) induced a significant decrease in cell viability of only 28%. Finally, at 72 h, the cell viability was significantly reduced at 50 (*p* = 0.0027), 100 (*p* < 0.0001), and 200 ppm (*p* < 0.0001), exhibiting percentages of viable cells of 72%, 57%, and 44%, respectively. These results suggest that the enhanced cytotoxicity observed with Fe_3_O_4_@SiO_2_/OXA in glioblastoma cells is due to the adsorption of oxaliplatin onto the nanoparticle surface, which potentiates the effect of the unconjugated oxaliplatin.

To validate the cytotoxicity of the magnetic core and SiO_2_ coating, an MTT assay was performed on the T98G and BHK-21 cell lines in the presence of Fe_3_O_4_ and Fe_3_O_4_@SiO_2_ nanoparticles (Fig. 12). The Fe_3_O_4_ nanoparticles did not exhibit cytotoxicity at the tested concentrations and time (IC_50_ at 72 h) in either cell line. They significantly increased the cell viability (*p* < 0.0001). Furthermore, the Fe_3_O_4_@SiO_2_ complex maintained a cell viability percentage like that of the untreated cells in T98G cells, while it caused a slight reduction (by 17%) in BHK-21 cells, a non-tumoral cell line (*p* < 0.0056). Previous studies have reported that iron oxide nanoparticles generally exert low cytotoxicity, often increasing reactive oxygen species (ROS) levels and lipid peroxidation.^38^ However, other studies have found no such effects, suggesting that their impact on cell viability depends on nanoparticle dose, coating, size, and cell type.^39^ Our findings confirmed the biocompatibility of the synthesised nanoparticles and showed that the magnetic core and SiO_2_ coating did not influence the high cytotoxicity of the Fe_3_O_4_@SiO_2_/OXA complex in glioblastoma cells.

GBM is a highly heterogeneous and aggressive brain tumor, and no single cell line can fully reproduce its biological diversity. In this study, the T98G cell line was selected as a validated and clinically relevant GBM model, particularly suitable for investigations focused on metabolic reprogramming and therapy resistance. T98G cells replicate the glycolytic activity commonly observed in primary GBM, display intrinsic resistance to temozolomide (TMZ), the standard chemotherapeutic agent used in GBM treatment, and share phenotypic and transcriptional characteristics with other widely used glioblastoma models (U87MG, LN229, A172), including hypoxia-driven adaptations and stem-like features associated with tumor recurrence. Comparative analyses among several GBM cell lines (T98G, U87MG, LN229, U251MG) have demonstrated that T98G retains the main molecular hallmarks of GBM, supporting its use in mechanistic and preclinical studies. Moreover, T98G cells exhibit a clinically relevant resistance phenotype characterized by elevated TMZ IC_50_ values, radioresistance, and a distinct oxidative mitochondrial profile linked to metabolic resilience. These properties make T98G a suitable and translationally relevant model for evaluating nanoparticle-based drug delivery systems designed to overcome chemoresistance and metabolic adaptation in glioblastoma.

In this study, the Fe_3_O_4_@SiO_2_/OXA nanocomposite was conceived as a theranostic platform whose biological activity is expected to depend primarily on tumor microenvironment-specific factors, including local accumulation, targeted delivery, and controlled oxaliplatin release. For this reason, cytotoxicity assays were focused on glioblastoma cells rather than non-tumoral fibroblasts, as the latter do not recapitulate the microenvironmental conditions that govern the selective action of the nanoplatform. The cytotoxicity of oxaliplatin in non-malignant cells, such as fibroblasts, has been widely reported in the literature, consistently showing lower susceptibility compared to tumor cells.^40–44^ Similar behavior has also been observed in related oxaliplatin-based nanocarrier systems, such as Oxa@MIL-100(Fe), which exhibited minimal effects in normal cell lines (*e.g.*, GES-1) while maintaining marked cytotoxicity in cancer models.^44–48^ These findings support the notion that tumor selectivity in such systems arises mainly from microenvironment-dependent mechanisms rather than intrinsic differences in cell-type sensitivity. Nevertheless, to further characterize the biosafety and selectivity profile of the Fe_3_O_4_@SiO_2_/OXA nanocomposite, future studies will include cytotoxicity assessments in astrocytes and other non-tumoral brain cells.

### 3.3 Intracellular localisation of Fe_3_O_4_@SiO_2_/OXA

**Fig. 13:**
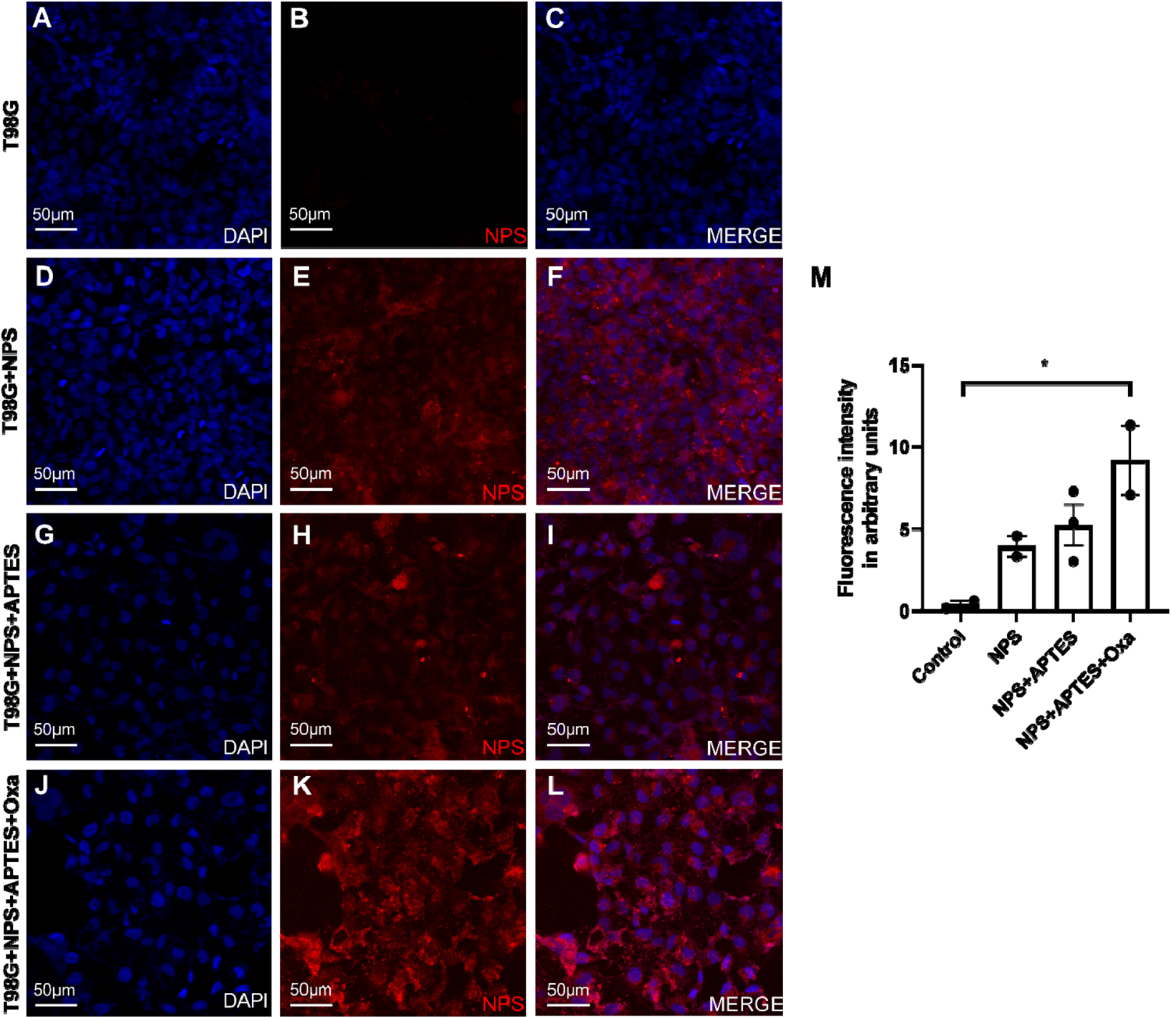
Immunofluorescence of T98G cells incubated with Fe_3_O_4_@SiO_2_ functionalised nanoparticles with oxaliplatin. (A–C) Cells without nanoparticles, (D–F) Fe_3_O_4_, (G–I) Fe_3_O_4_@SiO_2_, and (J–L) Fe_3_O_4_@SiO_2_ nanoparticles functionalised with oxaliplatin. Cell nuclei were stained with DAPI (blue), and nanoparticles were labelled with rhodamine (red). The bar graph (M) shows the relative fluorescence intensity of the nanoparticles under experimental conditions. Data are presented as mean ± standard deviation of the mean (SEM) (*n* = 3). Data were analysed using a one-way ANOVA with a Tukey post-hoc test (*p < 0.05).

As the Fe_3_O_4_@SiO_2_/OXA significantly increased the cytotoxic effect of the oxaliplatin alone, the cellular localisation of the nanocomposite and the intracellular changes induced after treatment for 72 h were determined. Fluorescence images revealed significant differences in the distribution and the number of rhodamine-labelled nanoparticles among the experimental groups (Fig. 13). T98G cells in the control group (Fig. 13A–C) exhibited no fluorescence in the rhodamine channel (Fig. 13B). In the Fe_3_O_4_-treated group, moderate intracellular fluorescence was observed (Fig. 13D–F), indicating successful internalisation of the nanoparticles. No apparent changes in cell morphology or population density were detected. Cells exposed to Fe_3_O_4_@SiO_2_ showed a more pronounced perinuclear accumulation of nanoparticles (Fig. 13G–I). Treatment with Fe_3_O_4_@SiO_2_/OXA resulted in a more prominent accumulation of nanocomposite in the perinuclear region (Fig. 13J–L), accompanied by a significant reduction in cell number, consistent with the expected cytotoxic effect of oxaliplatin. Quantitative analysis of fluorescence intensity corroborated these observations, showing a considerable increase (*p < 0.05) in the fluorescent signal of the oxaliplatin-treated group compared to the other groups, which correlates with the observed reduction in cell density (Fig. 13M).

**Fig. 14:**
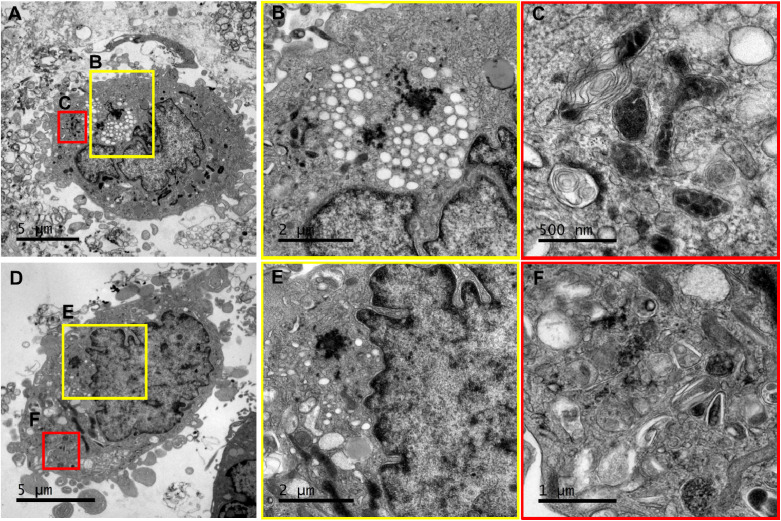
Transmission electron micrographs of T98G cells incubated with Fe_3_O_4_@SiO_2_ nanoparticles functionalised with oxaliplatin. T98G cells were exposed to nanoparticles for 72 hours. (A) Overview of the cell (scale = 5 µm); (B) magnification of the area marked by the yellow square in (A), showing agglomerated nanoparticles and vesicles near the nucleus (scale = 2 µm); (C) magnification of the area marked by the red square in (A), highlighting mitochondria with altered morphology (scale = 500 nm); (D) overview of the cell, scale = 5 µm; (E) magnification of the area marked by the yellow square in (D), displaying agglomerated nanoparticles near the nucleus (scale = 2 µm); (F) magnification of the area marked by the red square in (D), showing nanoparticles inside the vesicles in the cytoplasm (scale = 1 µm).

Additionally, TEM images of T98G glioblastoma cells treated with Fe_3_O_4_@SiO_2_/OXA for 72 h were taken (Fig. 14). Fig. 14A provides a panoramic view of a cell, revealing multiple areas of high electron density corresponding to the internalised nanocomposite and regions rich in intracellular vesicles distributed throughout the cytoplasm. In the enlarged view of this region, nanoparticle agglomerates are visible in a highly vesiculated area near the perinuclear zone (Fig. 14B). Fig. 14C shows structural alterations in the mitochondria, including swelling and disorganisation of the internal structure. Fig. 14D presents another cell where internalised nanoparticles are seen aggregated in the cytoplasm within the perinuclear zone, with additional nanoparticles encapsulated in vesicular structures (Fig. 14E and F).

Some authors have proposed that nanoparticles are internalised *via* a receptor-mediated endocytosis pathway, entering the cell through vesicles and fusing with lysosomes.^40^ In line with those findings, our observations confirm the cellular internalisation of the nanocomposite and suggest that the uptake of the nanocomposite may primarily involve the endocytic pathway, as indicated by images of Fe_3_O_4_@SiO_2_/OXA nanoparticles enclosed in intracellular vesicles. Interaction with lysosomes may facilitate the direct release of the drug into the intracellular environment. This release may be pH-dependent or triggered by hydrolytic enzymes, potentially enhancing the drug's effects compared to when it is internalised alone, as it could enter the cell nucleus more efficiently.^40–42^

However, the presence of nanoparticle agglomerates in the cytoplasm, not enclosed within membrane-bound structures, suggests that, in addition to the endocytic pathway, other processes may contribute to the release of nanoparticles into the cytoplasm or their internalisation *via* alternative mechanisms. Therefore, further studies are needed to deepen our understanding of the mechanism of action of oxaliplatin-functionalized nanoparticles in glioblastoma cells.

Crossing the blood–brain barrier (BBB) remains one of the most significant challenges in developing effective therapies for glioblastoma multiforme (GBM). Several studies have demonstrated that iron oxide-based nanostructures possess intrinsic properties that enable their translocation across the BBB and facilitate the brain delivery of therapeutic agents. For example, superparamagnetic iron oxide nanoparticles have been shown to markedly enhance the cerebral bioavailability of small molecules, such as quercetin, by up to tenfold compared with their free form, confirming their ability to traverse the BBB and act as brain-targeted carriers.^49^

Additional evidence from preclinical GBM models indicates that sub-5 nm magnetic nanoparticles exhibit superior BBB penetration and intratumoral accumulation, primarily due to their ultrafine size and the enhanced permeability and retention (EPR) effect. Likewise, lipid-coated and BBB-stealth iron oxide nanocomposites have demonstrated selective targeting of glioblastoma tissue and potent antitumor efficacy validated through MRI and bioluminescence imaging.

In the present work, the Fe_3_O_4_@SiO_2_/OXA nanocomposite maintained a spheroidal morphology following silica coating and oxaliplatin functionalization, with a controlled average size of 13–14 nm as determined by transmission electron microscopy. Nanoparticles within this size range, particularly those below 50 nm and with a spherical geometry, are known to exhibit enhanced cellular uptake and improved penetration across biological barriers, including the BBB. Collectively, these characteristics, together with prior evidence supporting the BBB permeability of iron oxide-based systems, suggest that the Fe_3_O_4_@SiO_2_/OXA nanoplatform holds realistic potential for effective brain delivery upon optimization of parameters such as particle size, surface functionalization, and biomimetic coating.

## 4. Conclusions

The present research demonstrates that the nanostructures produced by coprecipitation assisted by direct ultrasound exhibited an initial size of 12 nm. Upon coating with SiO_2_, the size increased to 13 nm, and further growth to 14 nm was observed after the physisorption of the drug oxaliplatin. Based on the measured XRD patterns, the crystal structure of the nanoparticles corresponds to the reverse spinel and magnetite phases. FTIR spectra confirmed the presence of a SiO_2_ coating and indicated the presence of vibrations associated with the drug in the nanocomposite. UV-Vis spectroscopy, which showed that the drug's concentration changes in the presence of the nanocomposite, further corroborated these findings. The results from VSM indicate that the nanoparticles exhibit superparamagnetic behaviour. The SiO_2_ coating induces a shielding effect but does not affect the superparamagnetic properties, allowing for their potential application in cancer therapeutics.^50^

The results of this study demonstrate that Fe_3_O_4_@SiO_2_/OXA nanocomposites significantly enhance the cytotoxicity of oxaliplatin in T98G glioblastoma cells. This finding suggests that the adsorption of oxaliplatin onto the nanoparticle surface potentiates the drug's cytotoxic effect, enhancing its therapeutic potential. Moreover, this research confirmed the biocompatibility of the Fe_3_O_4_@SiO_2_ nanoparticles, as neither the magnetic core (Fe_3_O_4_) nor the SiO_2_ coating exhibited significant cytotoxicity in the T98G or BHK-21 cell lines, supporting their potential for safe application in targeted cancer therapies. Fluorescence imaging and TEM analysis revealed that the Fe_3_O_4_@SiO_2_/OXA nanocomposite is successfully internalised into glioblastoma cells, with nanoparticles accumulating in the perinuclear region and inducing structural changes in organelles such as the mitochondria. These findings support the hypothesis that the nanocomposite is taken up primarily *via* an endocytic pathway, with the potential for pH- or enzyme-mediated drug release within the cell, which may contribute to the enhanced cytotoxic effect. Despite the successful internalisation and potent therapeutic effects of Fe_3_O_4_@SiO_2_/OXA, the presence of nanoparticle agglomerates in the cytoplasm suggests that additional internalisation mechanisms beyond endocytosis may be at play. Further studies are necessary to fully elucidate the intracellular mechanisms and optimise the design of these nanocomposites for improved therapeutic efficacy against glioblastoma and other cancers.

The silica coating plays a crucial role in enhancing the biological performance of the nanocomposite. The SiO_2_ shell improves the colloidal stability and biocompatibility of the Fe_3_O_4_ nanoparticles, prevents aggregation, and provides abundant surface hydroxyl groups that facilitate oxaliplatin adsorption and sustained release. These features promote a more efficient interaction with the cell membrane, resulting in increased intracellular drug delivery and enhanced cytotoxicity. Moreover, inhibition studies using pathway-specific endocytic blockers (such as chlorpromazine and genistein) revealed a significant reduction in nanoparticle internalization, confirming that Fe_3_O_4_@SiO_2_/OXA nanocomposites are primarily taken up by T98G cells through an endocytic pathway.

## Conflicts of interest

There are no conflicts to declare.

## Data Availability

The datasets generated and analyzed during this study are available from the corresponding author, Javier Rincón, upon reasonable request.
